# How to outsmart NK cell tolerance

**DOI:** 10.1080/2162402X.2015.1016708

**Published:** 2015-04-02

**Authors:** Grzegorz Terszowski, Christian Klein, Laurent Schmied, Martin Stern

**Affiliations:** 1 Department of Biomedicine; University Hospital Basel; Basel, Switzerland; 2 Roche Pharma Research and Early Development; Roche Innovation Center Zurich; Schlieren, Switzerland

**Keywords:** NK cell tolerance, KIR, HLA, ADCC, rituximab, obinutuzumab, lirilumab, CD20

## Abstract

Immune tolerance induced by regulatory mechanisms is an integral and fundamental part of immunity. In therapeutic settings, however, tolerance may significantly limit efficacy. Here, we summarize possible strategies to enhance therapeutic antibody dependent cellular cytotoxicity by overcoming NK cell tolerance.

Along with their potential for strong cytotoxic responses without previous priming, the common expression of the low-affinity immunoglobulin gamma Fc region receptor III-A (FcγR3A) makes natural killer (NK) cells potent effectors of therapeutic antibody dependent cellular cytotoxicity (ADCC). Nevertheless, ADCC efficacy is limited by NK cell intrinsic and extrinsic regulatory mechanisms. In particular, the interaction between inhibitory killer-cell immunoglobulin-like receptors (KIR) and their HLA Class I ligands is fundamental to NK cell development and tolerance. In a process called “licensing”, only NK cells expressing inhibitory receptors able to interact with self-HLA molecules gain full functional capacity. Potentially autoreactive NK cells carrying KIR that lack a cognate HLA ligand stay hypo-functional.

Rituximab – a chimeric anti-CD20 antibody – is a standard component of regimens used to treat B-cell lymphoma. How NK cell function in rituximab-induced ADCC is affected by regulatory mechanisms has not yet been completely defined.^1^ We recently addressed the question of how KIR/HLA interactions influence rituximab-induced ADCC, and showed that the advantage of the full functional potential of licensed NK cells is compensated by the inhibitory KIR signal, if target cells express cognate HLA (**Fig. 1A**).^2^ In line with the concept of unlicensed NK cells being the strongest ADCC effector cells, we observed that killing efficiency correlated positively with the percentage of unlicensed cells. Considering that in Caucasians approximately 30 percent of individuals carry all HLA ligands to the 3 relevant inhibitory KIR receptors (KIR2DL1, KIR2DL2/3, and KIR3DL1), the benefit of anti-CD20 therapy in such patients may be strongly limited by NK cell tolerance. Our *in vitro* data were recently confirmed by the analysis of follicular lymphoma patients treated with rituximab, which showed that progression-free survival (PFS) decreases with the number of viable KIR/HLA interactions.^3^ These data indicate a need for strategies to overcome the negative impact of KIR/HLA interactions in order to enhance the efficacy of therapeutic antibodies.

**Figure 1.**
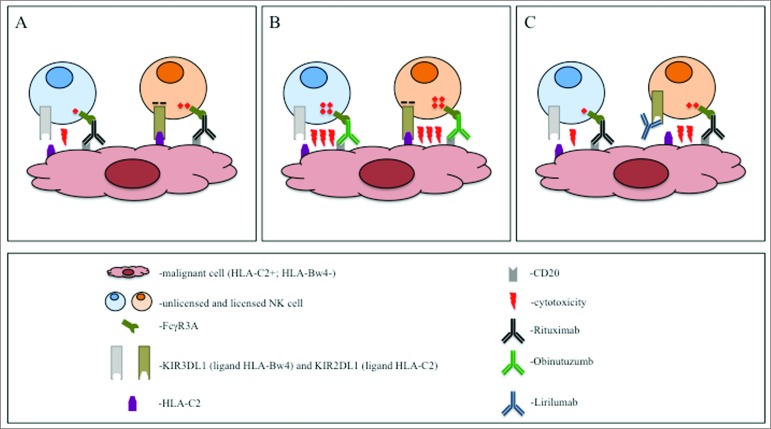
Strategies to induce NK-cell-driven antibody dependent cell cytotoxicity (ADCC) against lymphoma. CD20^+^ malignant B cells in an HLA-C2^+^, Bw4^−^ individual are treated with anti-CD20-antibodies. The presence of the HLA-C2 and the lack of HLA-Bw4 defines the KIR2DL1^+^ natural killer (NK) cell as licensed and KIR3DL1^+^ as unlicensed, respectively. (**A**) The rituximab-induced activating signal is inhibited by the KIR2DL1/HLA-C2 interaction in the licensed NK cell. The activation in the KIR3DL1^+^ NK cell cannot be inhibited because of the lack of the HLA-Bw4 on the malignant cell. However, its activity is compromised by its unlicensed status. (**B**) The stronger activation induced by obinutuzumab overrides the inhibitory signal in the licensed KIR2DL1^+^ cell. In addition, the activation is superior to the unlicensed status of the KIR3DL1^+^ cell. (**C**) Due to the block of the KIR2DL1/HLA-C2 interaction by lirilumab, the licensed KIR2DL1^+^ cell can be activated with rituximab. Lirilumab does not react with the KIR3DL1. Therefore, the activity of the unlicensed KIR3DL1^+^ cell is not influenced.

The observation that rituximab had greater clinical efficacy in patients carrying the high-affinity FcγR3A^4^ led to the development of new anti-CD20 antibodies with modified Fc regions aiming to strengthen Fc/FcR interactions. One such antibody is obinutuzumab carrying an afucosylated glycoengineered Fc part, which increases the affinity to the FcγR3A receptor, and thereby enhances NK cell activation and killing efficiency.^5,6^ Based on increased response rates and prolonged PFS as compared to rituximab when given in combination with chlorambucil to patients with chronic lymphocytic leukemia, obinutuzumab was recently approved for this indication and is undergoing evaluation in other types of B-cell lymphoma.^7^

*In vitro*, obinutuzumab recruited more NK cells for ADCC and activated them more strongly than rituximab. Most notably, licensed and unlicensed cells practically did not differ in their level of activation and the activation was hardly influenced by the presence of cognate HLA KIR ligand on target cells. Importantly, multiple KIR/HLA interactions were necessary to decrease obinutuzumab-induced ADCC to the level achieved by rituximab.^2^ In line with these observations, target depletion was unaffected by both the percentage of unlicensed effector cells in the repertoire, and by the number of KIR ligands expressed on target cells. In summary, obinutuzumab can induce comparable percentage and quality of activation in all NK cells subpopulations, independent from the licensing status and the KIR/HLA interactions (**Fig. 1B**). These data suggest that in contrast to rituximab, obinutuzumab efficacy may not correlate with either the KIR or HLA genotypes, a hypothesis still awaiting clinical testing.

Another strategy to overcome the suppressive KIR/HLA interaction is based on antibody blockade. To this end, an antagonistic, non-depleting anti-KIRD2 antibody (lirilumab) has been developed. Lirilumab can block the KIR/HLA interaction in approximately half of NK cells (those expressing KIR2D receptors), increasing the number of activated immune effector cells *in vitro* as well as *in vivo*.^8^ However, anti-KIR antibody treatment does not augment the strength of activation, and will therefore not increase the level of activation per cell (**Fig. 1C**). The global blockage of the KIR function may also carry a potential risk of autoimmunity. However, clinical trials in patients with multiple myeloma report no evidence of autoimmune reactions.^9^ Preclinically, use of rituximab in combination with lirilumab increased ADCC efficacy both *in vitro* and in mouse models.^10^ While no clinical data are available on this combination so far, a phase-one studies is currently testing lirilumab in combination with elotuzumab (anti-CS1) in patients with multiple myeloma (NCT02252263).

The differences between the 2 presented strategies are too great to determine which one could be more beneficial. The advantage of the strategy represented by obinutuzumab is maximal percentage and level of activation of all FcγR3A-positive NK cells independently from their licensing status and the KIR/HLA interaction mediated by a single agent therapy. Fc enhancement can easily be incorporated into the development of new therapeutic antibodies. In contrast, lirilumab can be added to existing therapies in which the KIR/HLA interaction limits therapeutic benefits.
